# Rescue or murder? The effect of prey adaptation to the predator subjected to fisheries

**DOI:** 10.1002/ece3.70336

**Published:** 2024-12-04

**Authors:** Yangke Shang, Minoru Kasada, Michio Kondoh

**Affiliations:** ^1^ Graduate School of Life Sciences Tohoku University Sendai Japan

**Keywords:** coevolutionary dynamic, evolutionary murder, fisheries‐induced evolution, indirect evolutionary rescue, predator–prey system, selective harvest

## Abstract

The concept of “indirect evolutionary rescue” refers to the evolutionary adaptation of an interacting species that can save a focal species from extinction in an unfavorable environment. Although theories suggest that indirect evolutionary rescue may have essential impacts on catchments in the context of fisheries where artificial selection pressure from fishing can drive evolution, its generality and conditions remain uncertain. In this study, by investigating how prey adaptation affects the persistence of a predator subjected to selective harvest with an eco‐evolutionary predator–prey model, we find that prey adaptation tends to deteriorate (facilitate) predator persistence when predator's evolvability is high (low). In the system where the predator possesses high evolvability, selection by fisheries inhibits a predator's adaptation to prey, allowing the prey to escape predation by adaptation. Prey adaptation will affect predator persistence negatively, leading to evolutionary murder. Conversely, in the system where the predator's evolvability is low, the removal of predator individuals by fisheries relaxes predation pressure on prey, making the prey less defensive. Vulnerable prey affects predator persistence positively, resulting in indirect evolutionary rescue. The context‐dependent response of natural resources to fisheries identified in this study suggests that the eco‐evolutionary interplay should be considered for better natural resource management.

## Graphical abstract

Our model analysis reveals that prey adaptation can either rescue or murder the predator, depending on model settings. This context‐dependent nature stems from the relative strength of two impacts of fisheries, a decline in predator density and strengthened constraints on predator adaptation to the prey. The former renders the prey adaptation act as a rescuer, and the latter makes the prey adaptation serve as a murderer.

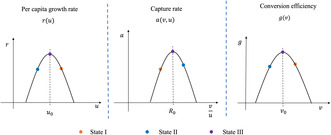

## 1 INTRODUCTION

With the advent of Anthropocene, human activities, such as climate change, human exploitation, and industrial pollution, have significantly altered natural environments and communities on a large scale (Handford et al., 1977; Klerks & Weis, 1987; Reid et al., 2005; Thomas et al., 2004). These alterations have led to populations experiencing declines in fitness and facing reduced population densities (Maynard Smith, 1989). In some cases, these deteriorations can even drive populations towards extinction. Increasing extinction risk makes the knowledge urgent for conservation. By now, it has been argued that adaptation to new environments, including migrating to proper regions, evolution, and learning can help populations to persist under unfavorable environments and mitigate the extinction risk (Carlson et al., 2014; Perry et al., 2005).

One of these well‐known adaptation processes is “evolutionary rescue” where a population improves its expected growth rate through evolutionary adaptation and avoids extinction in an unfavorable environment (Gomulkiewicz & Shaw, 2013; Gonzalez et al., 2013). In the context of multi‐species systems, evolution of one species can directly and indirectly affect the survival of other species. For example, in a predator–prey system, evolution of a prey species was shown to rescue its predator from extinction, a mechanism called indirect evolutionary rescue (Yamamichi & Miner, 2015). Indirect evolutionary rescue can occur if the prey defends against predation at the cost of its fitness, like the per capita growth rate or conversion efficiency. The underlying assumption for indirect evolutionary rescue in a predator–prey system is that the prey's per capita growth rate is consistently suppressed by predation, and external factors (e.g., environmental change, harvest, pollution) can alleviate this suppression by reducing the predator's density, while the declining defense of the prey leads to further rescue of the predator from extinction. Usually, these external factors can affect multi‐species systems at the demographic and/or at the adaptation level. For example, environmental changes taken as external factors impose additional mortality on the species (Cortez & Yamamichi, 2019; Goldberg & Friedman, 2021; Hermann & Becks, 2022; Raatz et al., 2019) or alter the optimal trait values that can maximize population growth rate (Mellard et al., 2015; Osmond et al., 2017). However, in fisheries, both processes may occur simultaneously and interact with each other. Fisheries are usually size‐selective, allocating fishing effort between individuals with different body sizes and leading to a distortion of the fitness landscape (Boukal et al., 2008; Conover & Munch, 2002; Jørgensen et al., 2009). In other words, this selective removal not only reduces population size, but also drives evolutionary adaptation of life‐history traits, referred to as “fisheries‐induced evolution (FIE)” (Heino et al., 2015).

In the field of fisheries, significant attention has been devoted to exploring the evolutionary response of target species to selective harvesting and their potential for recovery (Enberg et al., 2009; Heino et al., 2015; Walsh et al., 2006). However, in coevolutionary multispecies systems, fisheries can generate direct effects upon the target species that cascade through the system, inducing ecological and evolutionary responses of nontarget species. Afterwards, the response of nontarget species may give feedback on the target species. This feedback loop emphasizes the importance of identifying the influence of nontarget species upon target species, which have received relatively less consideration. This gap between the conservation of target species and the coevolutionary dynamics with nontarget species prompts our theoretical investigation into this topic.

To fill this gap, we constructed a mathematical model focusing on evolutionary changes in body size, as previous research has proposed that body size evolution leads to two distinct ecological consequences that may affect the eco‐evolutionary feedback loop. First, evolutionary changes in body size can affect life‐history strategies of the species (Bekoff et al., 1981; Kleiber, 1932; Peters, 1983; Reiss, 1988; Roff, 1992; Speakman, 2005). Body size has been widely used as a proxy of life‐history traits (Savage et al., 2004). For example, in fish species, evolutionary adaptation towards smaller body size usually results in earlier maturation and reduced fecundity per reproduction season (Roff, 1983; Uusi‐Heikkilä et al., 2010; Walsh et al., 2006), while larger body size will cause later maturation and increasing fecundity per reproduction season, but across fewer reproduction seasons (Boukal et al., 2008; Kuparinen et al., 2009). This implies the possibility that there is an optimal body size at which the lifetime reproduction output can be maximized (Einum & Fleming, 2000; Elgar, 1990; McEdward & Morgan, 2001; Roff, 1992). Secondly, alterations in body size can reshape the pattern and strength of interspecific relationships (Brose et al., 2006; Emmerson & Raffaelli, 2004; Renneville et al., 2016; Sinclair et al., 2003). The evolutionary adaptation of body size within predator–prey systems follows distinct patterns. The axis of prey vulnerability may exhibit either a unidirectional or bidirectional nature, contingent upon the evolving traits' characteristics (Abrams, 2000). In cases where a higher trait value consistently enhances the predator's capture rate and reduces predation risk for the prey, such as in situations involving speed versus speed, toxin versus antitoxin, or weapon versus armor, the assumption of a unidirectional axis of prey vulnerability is appropriate (Frank, 1994; Mougi & Iwasa, 2010; Nuismer et al., 2007; Saloniemi, 1993; Sasaki & Godfray, 1999). This scenario has been theoretically exemplified in the work of Yamamichi and Miner (2015). Conversely, when prey can minimize their risk by possessing a trait value either larger or smaller than the most vulnerable trait determined by the predator's traits, it is more fitting to assume a bidirectional axis of prey vulnerability, such as body size as an evolving trait (Calcagno et al., 2010; Dieckmann et al., 1995; Doebeli, 1997; Gavrilets, 1997; Kopp & Gavrilets, 2006; Nuismer et al., 2005).

In this study, we aim to examine the role of prey evolutionary adaptations in the persistence of predators within fisheries using an eco‐evolutionary predator–prey model. To achieve our goal, we compare the differences in predator persistence between scenarios where the prey can evolve adaptively and scenarios where it cannot. We employ two extreme cases to illustrate contrasting mechanisms: one demonstrating evolutionary murder and the other showing indirect evolutionary rescue. Subsequently, we apply these mechanisms to explain the impact of prey evolutionary adaptation on predator persistence in more general cases. Through this analysis, we shed light on the potential effects of prey evolutionary adaptation in the face of predators suffering from selective harvest.

## 2 METHOD

### 2.1 Model

#### 2.1.1 Population dynamics

Our model is based on the Lotka–Volterra population dynamics model of the prey and predator. Prey population size (*x*) follows logistic growth with mortality increasing with predator population size (*y*) according to the Holling type I (linear) function. In addition to natural mortality, the predator population is subjected to fisheries‐induced mortality. In this model, body size is selected as the only evolving trait for the prey (*u*) and predator (*v*), which are defined by a quantitative trait model. The population dynamics can be described by the following equations:

(1) $$\frac{\mathrm{dx}}{\mathrm{dt}}=\left(r-cx-\mathrm{ay}\right)x$$

(2) $$\frac{\mathrm{dy}}{\mathrm{dt}}=\left(\mathrm{gax}-d-F\right)y$$

where *x* and *y* denote the prey and predator population sizes, respectively; *r* represents the per capita prey growth rate; *c* denotes the strength of density dependence; *a* represents the capture rate; *g* denotes the conversion efficiency for the predator; *d* represents the predator's natural mortality rate; and *F* represents the fishing mortality rate.

**FIGURE 1:**
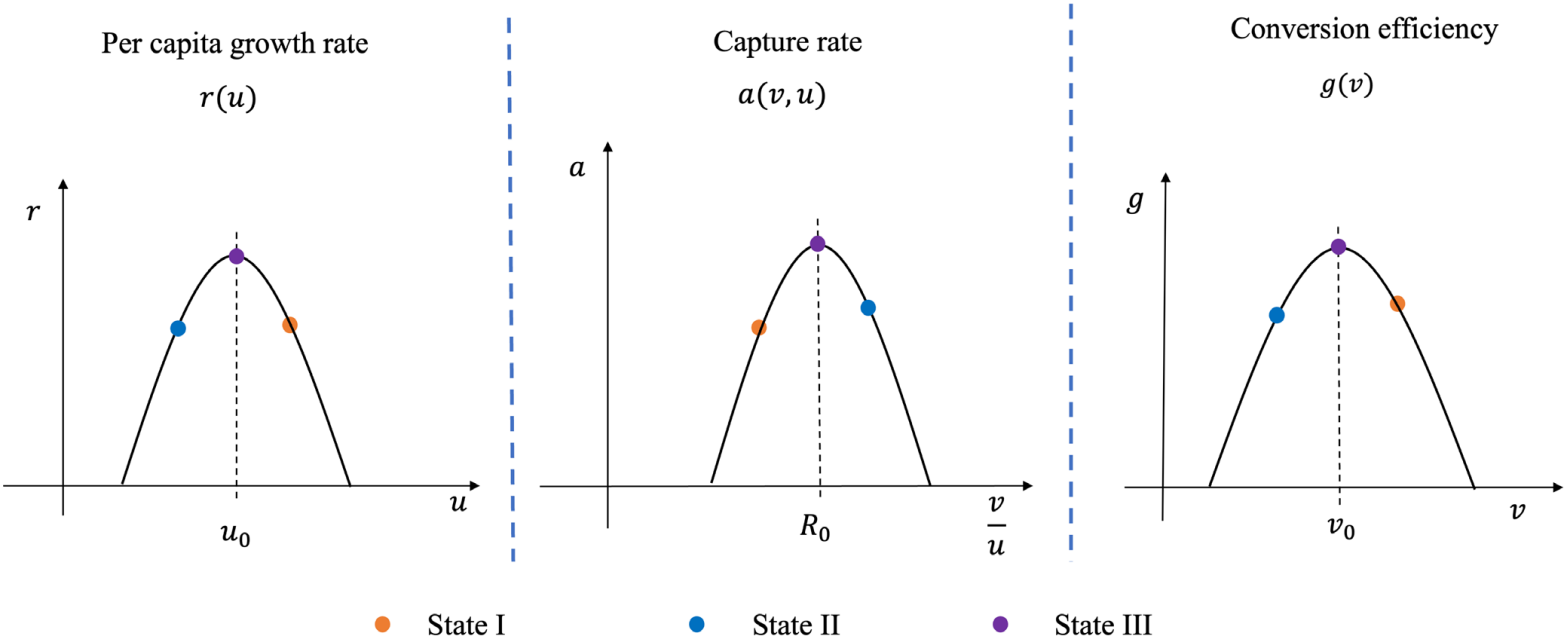
Schematic representation of the model setting and equilibrium states. Orange points denote State I, where $v^{*}>v_{0}$, $u^{*}>u_{0}$ and $\frac{v^{*}}{u^{*}}<R_{0}$. Blue points represent State II, characterized by $v^{*}<v_{0}$, $u^{*}<u_{0}$ and $\frac{v^{*}}{u^{*}}>R_{0}$. Purple points depict State III, where $v^{*}=v_{0}$, $u^{*}=u_{0}$ and $\frac{v^{*}}{u^{*}}=R_{0}$.

The parameters *r*, *a*, and *g* are functions of the body size for each species (Figure 1). Following earlier theoretical studies (Brown et al., 1993; Maiorana, 1990; Maurer, 1998; Mougi, 2012), we assume that there is an optimal body size that maximizes prey per capita growth rate and predator conversion efficiency. Correspondingly, the relationships between prey per capita growth rate (*r*) and prey body size (*u*), as well as predator conversion efficiency (*g*) and predator body size (*v*) can be assumed mathematically as: (1) $\frac{d^{2}r}{du^{2}}<0$, ${\left.\frac{\mathrm{dr}}{\mathrm{du}}\right|}_{u=u_{0}}=0$, $r\left(u_{0}\right)>0$ for prey, (2) $\frac{d^{2}g}{dv^{2}}<0$, ${\left.\frac{\mathrm{dg}}{\mathrm{dv}}\right|}_{v=v_{0}}=0$, $g\left(v_{0}\right)>0$ for predator, where $u_{0}$ and $v_{0}$ are the optimal body size for the prey and predator, which can maximize prey per capita growth rate (*r*) and predator conversion efficiency (*g*), respectively. The capture rate (*a*) is postulated as a function of body size ratio between the predator and prey. We assume that predators cannot utilize prey that are either too large, due to physical constraints on feeding (i.e., gape limitation), or too small, due to their low nutritional value. Therefore, the interaction strength should be maximized at some intermediate level of body size ratio (Brose, 2010). The body size ratio which can maximize the capture rate is termed “optimal body size ratio” here. Any deviations from the optimal body size ratio ($R_{0}$) will decrease capture rate (Brose, 2010; Cohen et al., 1993). Mathematically, the assumption can be depicted as: (3) ${\left.\frac{d^{2}a}{d{\left(\frac{v}{u}\right)}^{2}}<0,\frac{\mathrm{da}}{d\left(\frac{v}{u}\right)}\right|}_{\frac{v}{u}=R_{0}}=0,a\left(R_{0}\right)>0$.

We assume that fishing mortality is represented as a bell‐shaped function of body size, as expected in fisheries using gillnets, in which fish with a certain girth are captured with a higher probability than smaller fish that can slip through or larger fish that do not get far enough through the mesh to get stuck (Hamley, 1975) (Figure S1). By regulating mesh size, gillnets can target individuals with a specific range of body sizes. The gillnet harvest mortality is modeled as the following equation:

$$F=He^{-{\left(\frac{v-l_{\max}}{p}\right)}^{2}}$$

where *H* represents the harvest intensity, *v* denotes the predator body size targeted by fisheries, $l_{\max}$ corresponds to the body size at which the selectivity peaks, and *p* is the shape parameter. This function indicates that individuals with body sizes closer to $l_{\max}$ are more susceptible to fisheries.

#### 2.1.2 Dynamics of evolutionary adaptation

The dynamics of evolutionary adaptation of population mean body size (*u* and *v*) are modeled following the quantitative genetics approach derived in Lande (1976, 1982) and Abrams et al. (1993) with the following equations,

(3) $$\frac{\mathrm{du}}{\mathrm{dt}}=V_{x}\left(\frac{\mathrm{dr}}{\mathrm{du}}-\frac{\partial a}{\partial u}∙y\right)$$

(4) $$\frac{\mathrm{dv}}{\mathrm{dt}}=V_{y}\left(\frac{\mathrm{dg}}{\mathrm{dv}}∙\mathrm{ax}+\frac{\partial a}{\partial v}∙\mathrm{gx}-\frac{\mathrm{dF}}{\mathrm{dv}}\right)$$

where $V_{x}$ and $V_{y}$ represent the additive genetic variation in the prey and predator populations, respectively. These variations determine the speed of evolutionary adaptation. For simplicity, we assume a constant speed of adaptation. Equations (3) and (4) indicate that the rate of adaptive change in the traits is proportional to the selection gradient. If the selection gradient is positive (negative), it drives the species towards a larger (smaller) body size by pushing the mean trait value in that direction. Considering that the equations (3) and (4) can not only depict the changes in the traits resulting from genetic evolution, but also illustrate the changes due to phenotypic plasticity (Abrams et al., 1993), the term of “adaptation” is adopted to illustrate any of these mechanisms.

In this research, by regulating the value of $l_{\max}$, specific predator individuals can be targeted by fisheries (Figure S1). When the predator body size (*v*) is smaller than ($l_{\max}$), the individuals with larger body size will suffer from higher fishing mortality, and selective harvest tends to drive the predator to become smaller. This is indicated by the positive value of ${\left.\frac{\mathrm{dF}}{\mathrm{dv}}\right|}_{v<l_{\max}}$ (Figure S1b). Conversely, when predator body size (*v*) is larger than ($l_{\max}$), the selective harvest tends to drive the species to become larger. This is reflected by the negative value of ${\left.\frac{\mathrm{dF}}{\mathrm{dv}}\right|}_{v>l_{\max}}$, indicating that individuals with smaller body size will be subjected to higher fishing mortality (Figure S1b). Considering the symmetry between the small‐harvested patterns ($l_{\max}<v$) and large‐harvested patterns ($l_{\max}>v$), only will the large‐harvested patterns be focused on in this study.

These four differential equations, (1), (2), (3), and (4), capture the coupled coevolutionary and ecological dynamics between a prey species and a predator species suffering from fisheries. The response of the predator–prey system to fisheries will be examined based on this eco‐evolutionary model.

### 2.2 Analysis strategy

By combining fundamental eco‐evolutionary predator–prey systems with a selective harvest component, we can examine how prey adaptation affects predator persistence in fisheries. To highlight the influence of prey adaptation, the scenario of nonevolvable prey is adopted for comparison ($V_{x}=0$). Once fisheries are imposed, the prey's body size remains fixed at its pre‐harvest equilibrium ($u_{e}^{*}$) in the nonevolvable prey scenario. Conversely, in the evolvable prey scenario ($V_{x}\neq 0$), the prey can adaptively evolve to fisheries.

In this study, we assumed a unimodal relationship of prey per capita growth rate and predator conversion efficiency. The sharpness of these unimodal curves can be used to evaluate the evolvability of both prey and predator. A flat unimodal curve indicates high evolvability for the predator (or prey), resulting in intense predation on prey (or weak predation). Conversely, a steep unimodal curve constrains the predator's (or prey's) evolvability to maximize predation (or minimize predation). Thus, the evolvability of the prey and predator can be represented by the value of $\frac{d^{2}r}{du^{2}}$ and $\frac{d^{2}g}{dv^{2}}$, ranging from −∞ to 0. When $\frac{d^{2}r}{du^{2}}$ and $\frac{d^{2}g}{dv^{2}}$ approach 0 (or −∞), the prey and predator exhibit high (or low) evolvability.

Two extreme cases are proposed based on the predator's evolvability to facilitate the interpretation of the underlying mechanism. In case 1, the predator possesses high evolvability, represented mathematically as $\frac{d^{2}g}{dv^{2}}=0$. Because this is upper boundary (UB) value of the range $\left(-\infty ,0\right)$, this case is termed the UB case. In case 2, the predator possesses low evolvability, denoted mathematically as $\frac{d^{2}g}{dv^{2}}\to -\infty$. Given that this is lower boundary (LB) value of the range $\left(-\infty ,0\right)$, this case is named the LB case. When $\frac{d^{2}g}{dv^{2}}$ takes any value within the range from −∞ to 0, it is considered the general case.

We did not consider other extreme cases based on the prey's evolvability. The reason is that in the case where the prey possesses high evolvability ($\frac{d^{2}r}{du^{2}}=0$), prey adaptation would result in the extinction of the predator. In the case where prey possesses low evolvability ($\frac{d^{2}r}{dr^{2}}\to -\infty$), it is impossible to investigate the effect of prey adaptation on predator density. Therefore, the consequence of prey evolvability will only be studied numerically.

### 2.3 Mathematical analysis and numerical simulations

The general mathematical condition for the role of prey adaptation in predator persistence can be identified without assuming specific functions for prey per capita growth rate (*r*), conversion efficiency (*g*) and predation (*a*). When fisheries target the predator, the maximum fishing mortality ($F_{\max}$) at which the predator's density reaches the threshold value for extinction ($y_{\mathrm{tv}}$) serves as an indicator to evaluate the role of prey adaptation. If prey adaptation increases (or decreases) $F_{\max}$, the prey acts as a rescuer (or murderer). We assume that the threshold density is proportional to the predator density at pre‐harvest equilibrium with a ratio *e*, resulting in $y_{\mathrm{tv}}=e∙\frac{r\left(u^{*}\right)}{a\left(u^{*},v^{*}\right)}\left(1-\frac{\mathrm{cd}}{g\left(v^{*}\right)a\left(u^{*},v^{*}\right)r\left(u^{*}\right)}\right)$. When the system reaches a new equilibrium under the influence of fisheries, with predator and prey population sizes given by $y^{*}=\frac{r\left(u^{*}\right)-cx^{*}}{a\left(u^{*},v^{*}\right)}$ and $x^{*}=\frac{d+F\left(v^{*}\right)}{g\left(v^{*}\right)a\left(u^{*},v^{*}\right)}$, the maximum fishing mortality will be $F_{\max}=\left[\frac{g\left(v^{*}\right)a\left(u^{*},v^{*}\right)r\left(u^{*}\right)}{c}-d\right]∙\left(1-e\right)$.

To visualize the outcome of mathematical analysis, we specify functions for prey per capita growth rate (*r*), conversion efficiency (*g*) and predation (*a*) as follows: $r=-m{\left(u-u_{0}\right)}^{2}+r_{0}$, $g=-s{\left(v-v_{0}\right)}^{2}+g_{0}$ and $a=-t{\left(\frac{v}{u}-R_{0}\right)}^{2}+a_{0}$. Here, *m*, *s* and *t* control the curvature of corresponding curves, while $r_{0}$, $g_{0}$ and $a_{0}$ represent the maximum prey per capita growth rate, maximum predator conversion efficiency and maximum capture rate, respectively. By regulating the value of *m* and *s*, species evolvability can be controlled.

In the UB case ($\frac{d^{2}g}{dv^{2}}=0$), the function of $g\left(v\right)$ is replaced by $g=g_{0}$, indicating that changes in predator body size do not affect the conversion efficiency, which allows the predator to evolve freely to maximize predation. In the LB case ($\frac{d^{2}g}{dv^{2}}\to -\infty$), the conversion efficiency is expressed as

$$g=\left\{\begin{array}{c} g_{0},\text{if}\;v=v_{0}\\ 0,\text{if}\;v\neq v_{0} \end{array}\right..$$

This depicts that any slight deviation in body size from the optimal body size results in such a dramatic decline in conversion efficiency that any slight change in body size is prohibited. In general cases, the parameters *s* and *m* take on varied values, representing different evolvability for both predator and prey (Figure S2). The relative change of predator density caused by prey adaptation ($y_{\mathrm{rel}}$), compared to the pre‐harvest predator density ($y_{e}^{*}$), is used as an indicator to evaluate the effect of prey adaptation. This is defined as $y_{\mathrm{rel}}=\frac{y_{H\_\mathrm{evo}}^{*}-y_{H\_\text{nonevo}}^{*}}{y_{e}^{*}}$, where $y_{H\_\mathrm{evo}}^{*}$ and $y_{H\_\text{nonevo}}^{*}$ represent predator density at post‐harvest equilibrium in the scenarios of evolvable prey and nonevolvable prey, respectively. The relative changes of prey density ($x_{\mathrm{rel}}$), prey trait ($u_{\mathrm{rel}}$) and predator trait ($v_{\mathrm{rel}}$) are defined in the same way to depict how the system responds to fisheries.

## 3 RESULTS

### 3.1 Preharvest equilibrium states and stability analysis

When the predator–prey system reaches equilibrium, trait values of prey and predator should hold the following conditions:
(5)

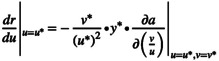



(6)

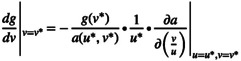




Our analytical analysis shows there are three equilibrium states (Figure 1, Appendix S1), illustrated as follows:

- State I: $v^{*}>v_{0}$, $u^{*}>u_{0}$ and $\frac{v^{*}}{u^{*}}<R_{0}$.
- State II: $v^{*}<v_{0}$, $u^{*}<u_{0}$ and $\frac{v^{*}}{u^{*}}>R_{0}$.
- State III: $v^{*}=v_{0}$, $u^{*}=u_{0}$ and $\frac{v^{*}}{u^{*}}=R_{0}$, implying a necessary condition $R_{0}=\frac{v_{0}}{u_{0}}$.

In State I and II, the benefit of prey adaptation from increasing the per capita growth rate is offset by the cost of increasing predation strength, and the benefit of predator adaptation from increasing conversion efficiency is counteracted by the cost of decreasing predation strength. In State III, the benefit of prey adaptation from decreasing predation strength is offset by the cost of the decline in its per capita growth rate. Given the symmetry between State I and State II, as well as the uniqueness of State III, we primarily focus on State I in this study. The other states can be analyzed similarly.

In these three equilibrium states, we assess the local stability of the system by linearizing the dynamics near the nontrivial equilibrium. The local stability is judged by whether the characteristic equation of the Jacobian matrix satisfies the Routh–Hurwitz criteria (Appendix S2). Due to the special nature of equilibrium points in the UB case from State I and in State III, their analytical stability condition can be obtained, which is:

$$\frac{V_{y}}{V_{x}}>\frac{R_{0}^{2}∙y^{*}-m*\frac{u_{0}^{2}}{t}}{g_{0}∙x^{*}}$$

In other cases, numerical analysis shows that the system is always stable in our study.

### 3.2 The effect of prey adaptation

#### 3.2.1 The UB case

In the UB case, the predator can evolve freely to maximize predation without any cost to its conversion efficiency ($g=g_{0}$ for any *v*). The equilibrium point for the evolutionary subsystem before fishing (denoted by the subscript “*e*”) can be attained, which is $\left\{u_{e}^{*}=u_{0},v_{e}^{*}=u_{0}R_{0}\right\}$. At this equilibrium point, the predator maximizes predation and conversion efficiency, while the prey experiences maximal predation strength at its maximum per capita growth rate.

When fisheries are imposed on the predator, due to fisheries selection targeting larger predator individuals (${\left.\frac{\mathrm{dF}}{\mathrm{dv}}\right|}_{v<l_{\max}}>0$), the predator will become smaller ($\frac{\mathrm{dv}}{\mathrm{dt}}<0$), resulting in a decline in capture rate ($\frac{\partial a}{\partial \left(\frac{v}{u}\right)}>0$). For the scenario of evolvable prey, the positive $\frac{\partial a}{\partial \left(\frac{v}{u}\right)}$ implies a negative $\frac{\partial a}{\partial u}$, leading to a positive $\frac{\mathrm{du}}{\mathrm{dt}}$ according to equation (3). When the system reaches the new equilibrium (denoted by the subscript “*H*”), predator adaptation will reach a new balance between fisheries selection (${\left.\frac{\mathrm{dF}}{\mathrm{dv}}\right|}_{v=v_{H}^{*}}>0$) and predation selection (${\left.\frac{\partial a}{\partial v}\right|}_{u=u_{H}^{*},v=v_{H}^{*}}>0$). Meanwhile, prey adaptation will satisfy the balance between predation selection (${\left.\frac{\partial a}{\partial u}\right|}_{u=u_{H}^{*},v=v_{H}^{*}}<0$) and the selection from its per capita growth rate (${\left.\frac{\mathrm{dr}}{\mathrm{du}}\right|}_{u=u_{H}^{*}}<0$). Therefore, at the new equilibrium point, $u_{H}^{*}>u_{0}$ and $\frac{v_{H}^{*}}{u_{H}^{*}}<R_{0}$ are obtained.

**FIGURE 2:**
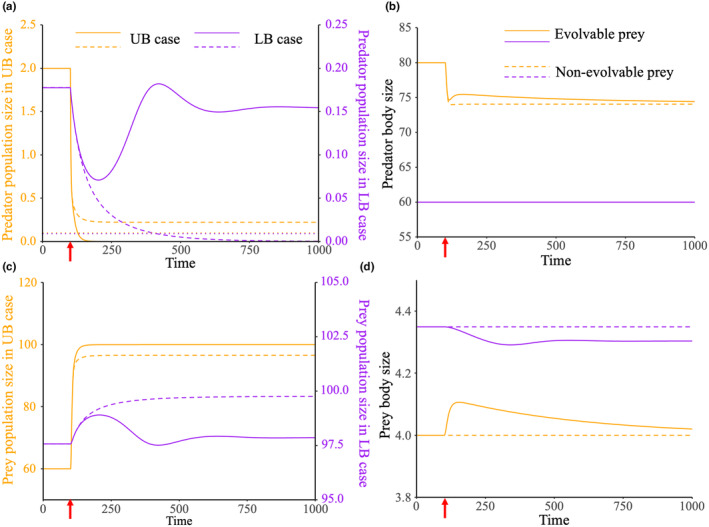
Response of the system to fisheries in numerical simulations for UB case and LB case. At time 100, fisheries are imposed upon the predator, indicated by red arrows. Orange and purple lines represent dynamics of the system in UB case and LB case, respectively. Dash lines and solid lines denote the scenarios of nonevolvable prey and evolvable prey, respectively. The dotted line represents the threshold density for predator extinction, set as 5% of predator density at pre‐harvest equilibrium. In panel b, dash line and solid line in LB case overlap due to the fixed predator body size. Parameter settings for all simulations: c=0.005, d=0.6, $g_{0}=0.1$, $a_{0}=0.1$, $r_{0}=0.5$, $u_{0}=4$, $v_{0}=60$, $R_{0}=20$, e=0.05, $V_{x}=0.1$ or 0 (for evolutionary and nonevolutionary scenarios, respectively), $V_{y}=15$, $l_{\max}=1.02∙v_{e}^{*}$, p=8. In the UB case: m=0.01, t=0.01, H=0.37; in the LB case, m=0.01, t=0.001, H=0.02, for better visualization without losing generality. $V_{x}=0.1$ and $V_{y}=15$ are chosen to ensure system stability under the reference of stability condition, without altering responses of the system to fisheries qualitatively. Initial value for simulations is $\left\{x=60,y=12,u=4,v=60\right\}$.

When $u>u_{0}$ and $\frac{v}{u}<R_{0}$, the maximum fishing mortality $F_{\max}=\left[\frac{g_{0}a\left(u^{*},v^{*}\right)r\left(u^{*}\right)}{c}-d\right]∙\left(1-e\right)$ is a decreasing function with respect to *u*, owing to the decreasing nature of $r\left(u\right)$ and $a\left(u,v\right)$. Therefore, in the UB case, prey adaptation will decrease $F_{\max}$, thereby inhibiting predator persistence. The extent of this inhibition depends on the increase in *u*, which is closely related to predator density according to equation (3). When fisheries are imposed upon the predator, prey adaptation can cause the predator density to fall below this threshold, leading to evolutionary murder (Figure 2).

The analytical findings are complemented by numerical simulations. After long‐term coevolution, the system reaches the evolutionary equilibrium point $\left\{u_{e}^{*}=u_{0}=4,v_{e}^{*}=u_{0}R_{0}=80\right\}$. Upon the imposition of large‐harvested fisheries on the predator, there is a sharp decline in predator population size, accompanied by a reduction in predator body size due to directional selection pressure from fisheries (Figure 2a,b). In response, prey population size exhibits a significant increase, while prey body size gradually enlarges in the scenario of evolvable prey (Figure 2c,d). Furthermore, prey adaptation causes a decrease in predator population density below the threshold density, leading to predator extinction (Figure 2a). The mechanism operates as follows: the predator with high evolvability maximizes predation, while the prey optimizes its fitness by achieving its optimal body size. However, when fisheries target the predator, selection pressure from fisheries constrains predator adaptation to maximize predation, offering the prey an opportunity to evade predation through adaptation. Consequently, the reduction in predation strength resulting from prey adaptation further undermines predator persistence in fisheries (Figure 2).

#### 3.2.2 The LB case

In the LB case, the conversion efficiency (*g*) is exceptionally sensitive to the predator's adaptation, such that any deviation from the optimal body size will result in g=0. Here, we assume $g=\left\{\begin{array}{c} g_{0},\left(v=v_{0}\right)\\ 0,\left(v\neq v_{0}\right) \end{array}\right.$, resulting in a three‐dimensional system depicted by equations (1), (2) and (3). The prey trait value at equilibrium is shown in equation (5), implying two possible equilibria: $\left\{u_{e}^{*}>u_{0},v_{e}^{*}=v_{0},\frac{v_{e}^{*}}{u_{e}^{*}}<R_{0}\right\}$ and $\left\{u_{e}^{*}<u_{0},v_{e}^{*}=v_{0},\frac{v_{e}^{*}}{u_{e}^{*}}>R_{0}\right\}$. These equilibria highlight the balance of prey adaptation between the prey per capita growth rate and predation strength. Specifically, the benefit of prey adaptation from one side must be counteracted by the cost from the other side.

Once the predator suffers from fisheries, its density decreases, subsequently weakening predation pressure. The prey responds to boost its per capita growth rate by becoming smaller under the first equilibrium and larger under the second equilibrium (equation 3). Considering the function $F_{\max}=\left[\frac{g_{0}a\left(u^{*},v^{*}\right)r\left(u^{*}\right)}{c}-d\right]∙\left(1-e\right)$, either a decline in *u* under the first equilibrium or an increase in *u* under the second equilibrium increases $F_{\max}$, because $r\left(u\right)$ and $a\left(u,v_{0}\right)$ are both decreasing (increasing) functions with respect to *u* under the first (second) equilibrium. Therefore, in the LB case, prey adaptation augments $F_{\max}$ and has the potential to facilitate predator density above the threshold value, leading to indirect evolutionary rescue (Figure 2).

The existence of two equilibria is highly parameter‐dependent. Under any equilibrium point, the prey adaptation imposes a positive effect upon predator persistence (Figure S3). Considering their symmetry, only the analytical analysis in the first equilibrium is complemented by numerical simulations. In simulations, the system reaches the equilibrium point at $\left\{u_{e}^{*}\approx 4.349519,v_{e}^{*}=60\right\}$ after long‐term coevolution. As the predator suffers from fisheries, the reduction in predator population size triggers an increase in prey population size and a decrease in prey body size in the scenario of evolvable prey. The decrease in prey body size intensifies predation, indirectly bolstering predator persistence (Figure 2).

#### 3.2.3 General cases

**FIGURE 3:**
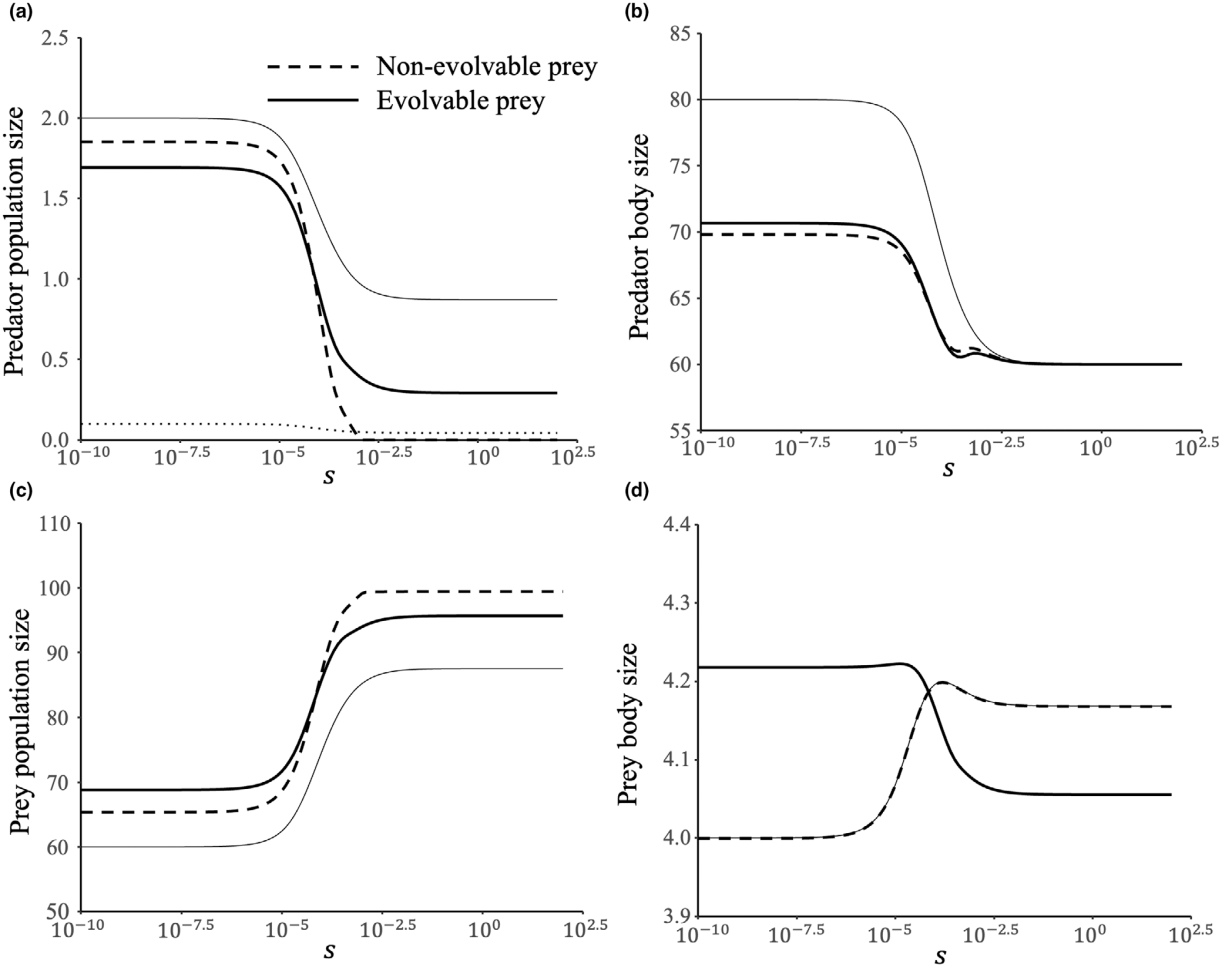
Effect of predator evolvability to responses of the system when the predator is subjected to fisheries. Thin solid lines represent traits and densities of the system at the pre‐harvest equilibrium. Thick dash lines and thick solid lines denote traits and densities at post‐harvest equilibrium under the scenarios of nonevolvable prey and evolvable prey, respectively. Parameters setting: m=0.1, t=0.001, H=0.1. Other parameters are the same as those in Figure 2.

We have demonstrated that prey adaptation can decrease the predator persistence in fisheries in the UB case and increase predator persistence in fisheries in the LB case. In more generalized cases where $\frac{d^{2}g}{dv^{2}}$ assumes an intermediate value, predator adaptation will alter conversion efficiency and predation simultaneously. In our numerical simulations, the value of *s* is utilized to depict the curvature of the expression for conversion efficiency. A smaller *s* indicates less sensitivity of conversion efficiency to predator adaptation (high evolvability), while a larger *s* signifies greater sensitivity (low evolvability). As s increases, the system's response to fisheries changes. When $s<10^{-6.0}$, the prey becomes larger in response to fishing pressure on the predator, which inhibits predator persistence, akin to the UB case. Conversely, when $s>10^{-2.0}$, the prey becomes smaller, which enhances predator persistence, corresponding to the LB case. The phase transition in the role of prey adaptation occurs within the range $10^{-6.0}<s<10^{-2.0}$ (Figure 3). Therefore, as predator evolvability decreases, the role of prey adaptation shifts from a murderer to a rescuer.

**FIGURE 4:**
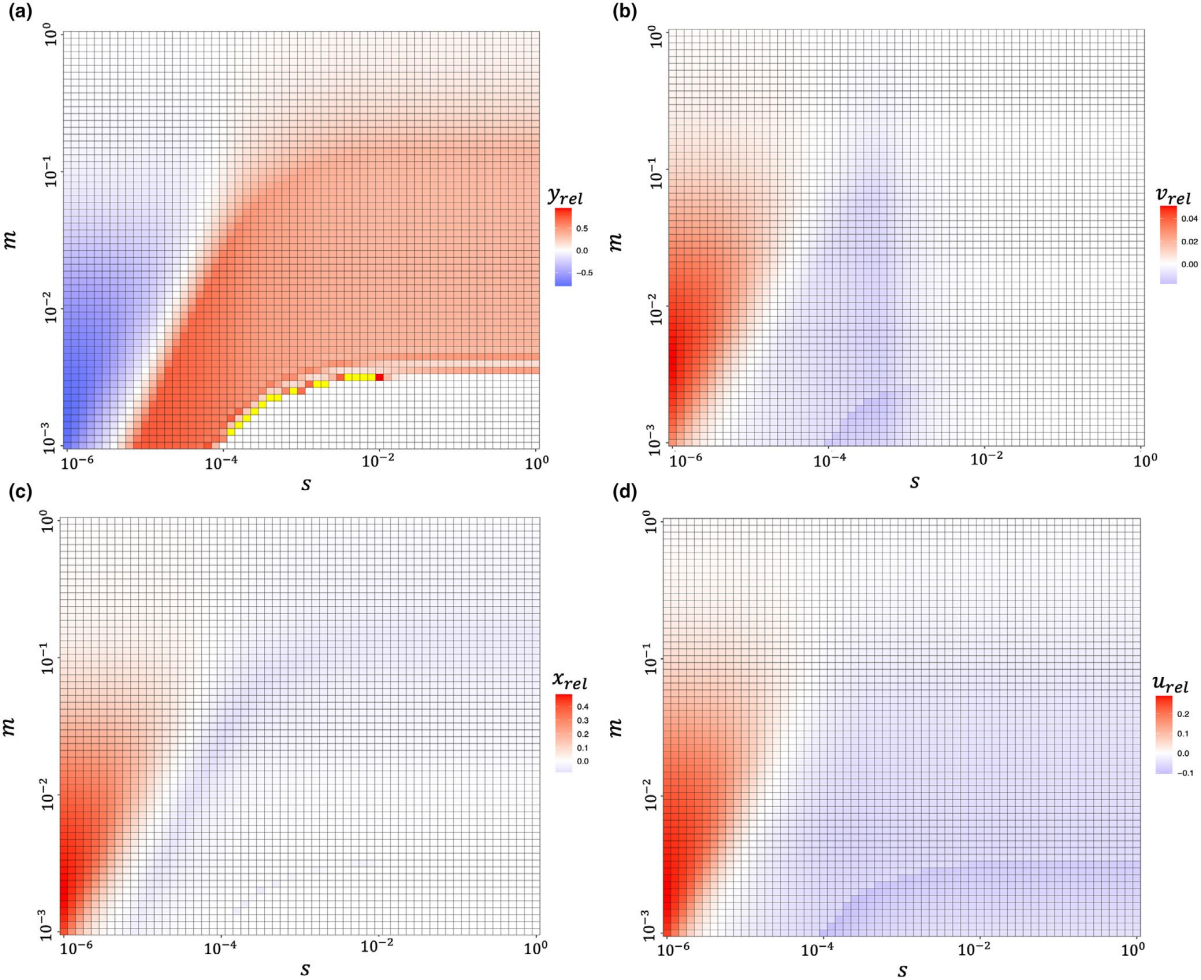
The relative change in traits and densities caused by prey adaptation under various combinations of prey and predator evolvability. The red regions represent that species densities or traits under scenarios of evolvable prey are larger than these under nonevolvable prey. The blue regions indicate instances of smaller densities or traits under scenarios of evolvable prey. In situations where the relative change exceeds 1, the corresponding regions are depicted in yellow based on consideration of visualization. Parameters settings: t=0.001, H=0.1. Other parameters are the same as those in Figure 2.

The above outcomes are robust across various levels of prey evolvability (Figure 4). Two distinct response patterns to fisheries can be observed. When the predator has high evolvability, prey's trait and density both increase compared to the scenario of nonevolvable prey, resulting in evolutionary murder. Conversely, when the predator's evolvability is low, prey's trait and density decrease, leading to indirect evolutionary rescue. In regions where prey evolvability is high and predator evolvability is low, the positive effect of prey adaptation is still present ($y_{H\_\mathrm{evo}}^{*}>y_{H\_\text{nonevo}}^{*}$), though very minimal. The minimal effect is due to the low predator density at post‐harvest equilibrium ($y_{H\_\mathrm{evo}}^{*}\approx 0$, $y_{H\_\text{nonevo}}^{*}\approx 0$), which results in $y_{\mathrm{rel}}\approx 0$. Additionally, the role of prey adaptation transitions under any levels of prey evolvability. As prey evolvability diminishes, the transition point also decreases with respect to predator evolvability. When prey evolvability is high, the transition is more dramatic, whereas with low prey evolvability, the transition becomes less pronounced.

## 4 DISCUSSION

The theory of indirect evolutionary rescue describes a mechanism whereby a population can endure in an unfavorable environment through the adaptation of an interacting and coevolving species, preventing its own extinction (Hermann & Becks, 2022; van Velzen, 2023; Yamamichi & Miner, 2015). However, our study suggests that indirect evolutionary rescue is conditional, and evolutionary murder occurs in some circumstances. We find that when a predator exhibits high evolvability, prey adaptation inhibits predator persistence in fisheries, assuming the role of a murderer. Conversely, in the system where a predator's evolvability is low, prey adaptation enhances predator persistence in fisheries, playing the role of a rescuer. As the predator's evolvability decreases, the function of prey adaptation gradually shifts from murderer to rescuer. The change of prey evolvability alters the position of transition occurrence quantitatively.

The distinct roles of prey adaptation stem from two key impacts of fisheries, causing a decline in predator density and imposing constraints on predator adaptation to the prey. These two features have the simultaneous capacity to impact the eco‐evolutionary dynamics of predator–prey systems (Edeline & Loeuille, 2021). The feature of density decline alters the ecological dynamics of the predator–prey system by decreasing the density of target species. Subsequently, the altered ecological dynamics induce changes in evolutionary dynamics through eco‐evolutionary feedback loops (Govaert et al., 2018; Lande et al., 2009; Lively, 2012). This pathway, where fisheries affect evolutionary dynamics through ecological dynamics, is termed the indirect pathway. Additionally, the feature of constraints in predator adaptation to the prey reshapes the fitness landscape and induces adaptation of the target species directly (Boukal et al., 2008; Conover & Munch, 2002; Heino et al., 2015; Jørgensen et al., 2009), which prompts the co‐evolution of its opponent. This pathway is termed the direct pathway. Both the indirect and direct pathways can simultaneously shape the evolutionary dynamics of the system.

In the indirect pathway, the decline in the predator density alleviates predation pressure on the prey. The relaxation in predation selection drives the prey to evolve in a manner that enhances its overall fitness, reinforcing its per capita growth rate at the expense of inadvertently intensifying predation (making it less adapted to the predator), as illustrated in the LB case. Therefore, the adaptation resulting from the feature of density decline makes the prey more vulnerable and promotes the capture rate, casting the prey in the role of a rescuer. Conversely, in the direct pathway, the feature of constraints in predator adaptation to the prey limits the predator's adaptability to maximize capture rate through adaptation. This limitation creates an opportunity for the prey to escape predation by adaptation (making it more adapted to the predator), rendering the prey less vulnerable and further dampening the capture rate, as illustrated in the UB case. Thus, the prey's adaptation arising from the feature of constraints in evolutionary potential acts as a murderer.

These two features can simultaneously affect the eco‐evolutionary dynamics of the predator–prey system through various pathways, leading to opposite consequences of prey adaptation on predator persistence. The dominance of one feature over the other is regulated by the species that faces fewer constraints on its adaptation from factors other than interspecific interaction. For instance, in the LB case where the prey faces fewer constraints on its adaptation from its per capita growth rate compared with the predator, the feature of density decline dominates, and causes prey adaptation to significantly rescue the predator. Conversely, in the UB case where the predator suffers from fewer constraints on its adaptation from its conversion efficiency compared with the prey, the feature of constraints in predator adaptation to prey takes precedence and renders prey adaptation considerably acting as a murderer of the predator. As the system transitions from the LB case to UB case, the evolutionary consequence of density decline gradually weakens, while the evolutionary consequence of constraints in adaptation is enhanced. Correspondingly, the role of prey adaptation shifts from a rescuer to a murderer.

Given that strengthened constraints on predator adaptation to prey lead to evolutionary murder, we hypothesize that evolutionary murder might result from other factors impeding the predator adaptation, such as the selective exploitation of species through activities like harvesting and fishing (Allendorf et al., 2008, 2013; Hauser et al., 2002; Sadler et al., 2023), an increased likelihood of inbreeding in small and fragmented populations (DiBattista, 2008; Keller & Waller, 2002; Willi et al., 2006), and the direction of selection arising from the absence or depletion of standing genetic variation (Araki et al., 2008; Benedict & Robinson, 2003; Kellermann et al., 2009). In these circumstances, inhibited predator adaptation may alter coevolutionary dynamics, thereby rendering evolutionary murder possible.

Recent investigations into indirect evolutionary rescue reveal the potential for its transformation into evolutionary murder in specific circumstances (Cortez & Yamamichi, 2019; van Velzen, 2023). While the concept of indirect evolutionary rescue holds promise for conservation scientists, our theoretical study suggests the context‐dependent nature of the effect of prey adaptation to their predators. Relying solely on the idea of indirect evolutionary rescue may lead to overly optimistic assessments, posing a risk of unexpected failures in conservation practices, especially when prey adaptation virtually plays the role of a murderer. The context‐dependent response of natural resources to fisheries suggests that the eco‐evolutionary interplay should be considered for better natural resource management.

## AUTHOR CONTRIBUTIONS

**Yangke Shang:** Conceptualization (equal); formal analysis (lead); investigation (lead); methodology (lead); software (lead); visualization (lead); writing – original draft (lead); writing – review and editing (lead). **Minoru Kasada:** Investigation (supporting); supervision (supporting); writing – original draft (supporting); writing – review and editing (supporting). **Michio Kondoh:** Conceptualization (equal); formal analysis (equal); funding acquisition (lead); investigation (equal); methodology (equal); project administration (equal); supervision (equal); writing – original draft (supporting); writing – review and editing (equal).

## FUNDING INFORMATION

Yangke Shang was supported by JST SPRING (grant number JPMJSP2114). Minoru Kasada was supported by a Grant‐in‐Aid for JSPS Fellows (grant number 19J00864). Michio Kondoh was supported by an internal grant for collaborative research in Graduate School of Sciences, Tohoku University; JSPS KAKENHI Grant (grant number 19H0564 and 21H05315) and the Environment Research and Technology Development Fund (grant number JPMEERF20214103) of the Environmental Restoration and Conservation Agency of Japan.

## CONFLICT OF INTEREST STATEMENT

The authors declare no conflict of interest.

## Supporting information


Data S1

## Data Availability

The simulation code and data supporting the findings of this study are openly available in Dryad at https://doi.org/10.5061/dryad.z08kprrmt.
