# Supplementary material for: Rescue or murder? The effect of prey adaptation to the predator subjected to fisheries
Source: Ecol Evol. 2024 Dec 4;14(12):e70336. doi: 10.1002/ece3.70336 (PMC11615651; doi:10.1002/ece3.70336)
Supplement: Supplementary file 1 — Data S1 [file ECE3-14-e70336-s001.docx]

**Supplementary Material belonging to**

Yangke Shang, Minoru Kasada, Michio Kondoh, *Rescue or murder? The effect of prey adaptation to the predator subjected to fisheries*

**Table of Contents**

[Figure S1: Fishing mortality and fishing selection strength of gillnets. 2](#_Toc176276207)

[Figure S2: The dependency of$\boldsymbol{r}\boldsymbol{(}\boldsymbol{u}\boldsymbol{)}$,$\boldsymbol{a}\boldsymbol{(}\boldsymbol{u}\boldsymbol{,}\boldsymbol{v}\boldsymbol{)}$ and $\boldsymbol{g}\boldsymbol{(}\boldsymbol{v}\boldsymbol{)}$ on traits. 2](#_Toc176276208)

[Figure S3: The impact of initial value setting to the final equilibrium point and effect of prey adaptation to predator persistence in LB case. 3](#_Toc176276209)

[Appendix S1: Model framework and equilibrium points 4](#_Toc176276210)

[Appendix S2: Stability analysis 6](#_Toc176276211)

[Stability evaluation method 6](#_Toc176276212)

[Stability conditions 7](#_Toc176276213)

# Figure S1: Fishing mortality and fishing selection strength of gillnets.


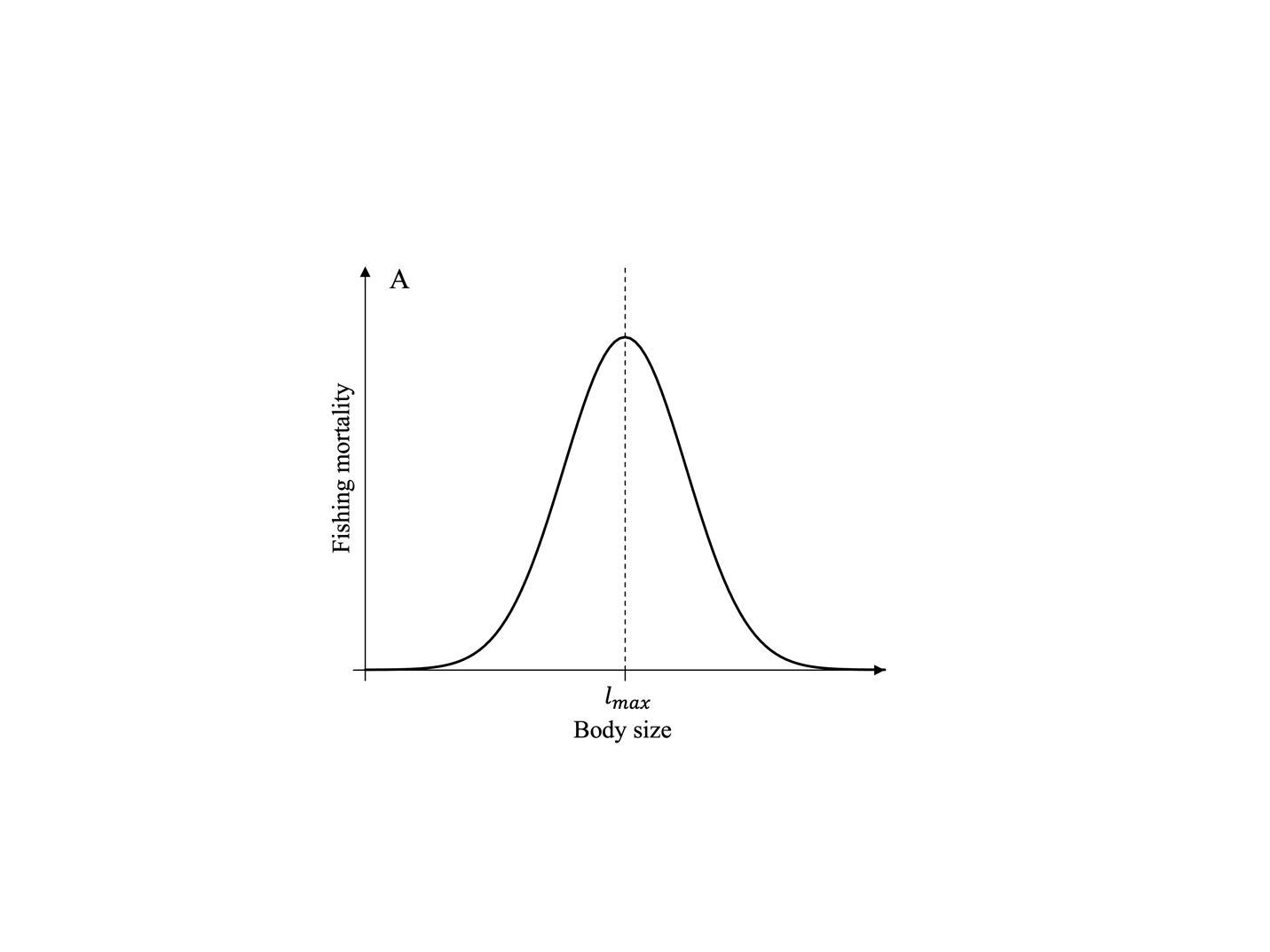

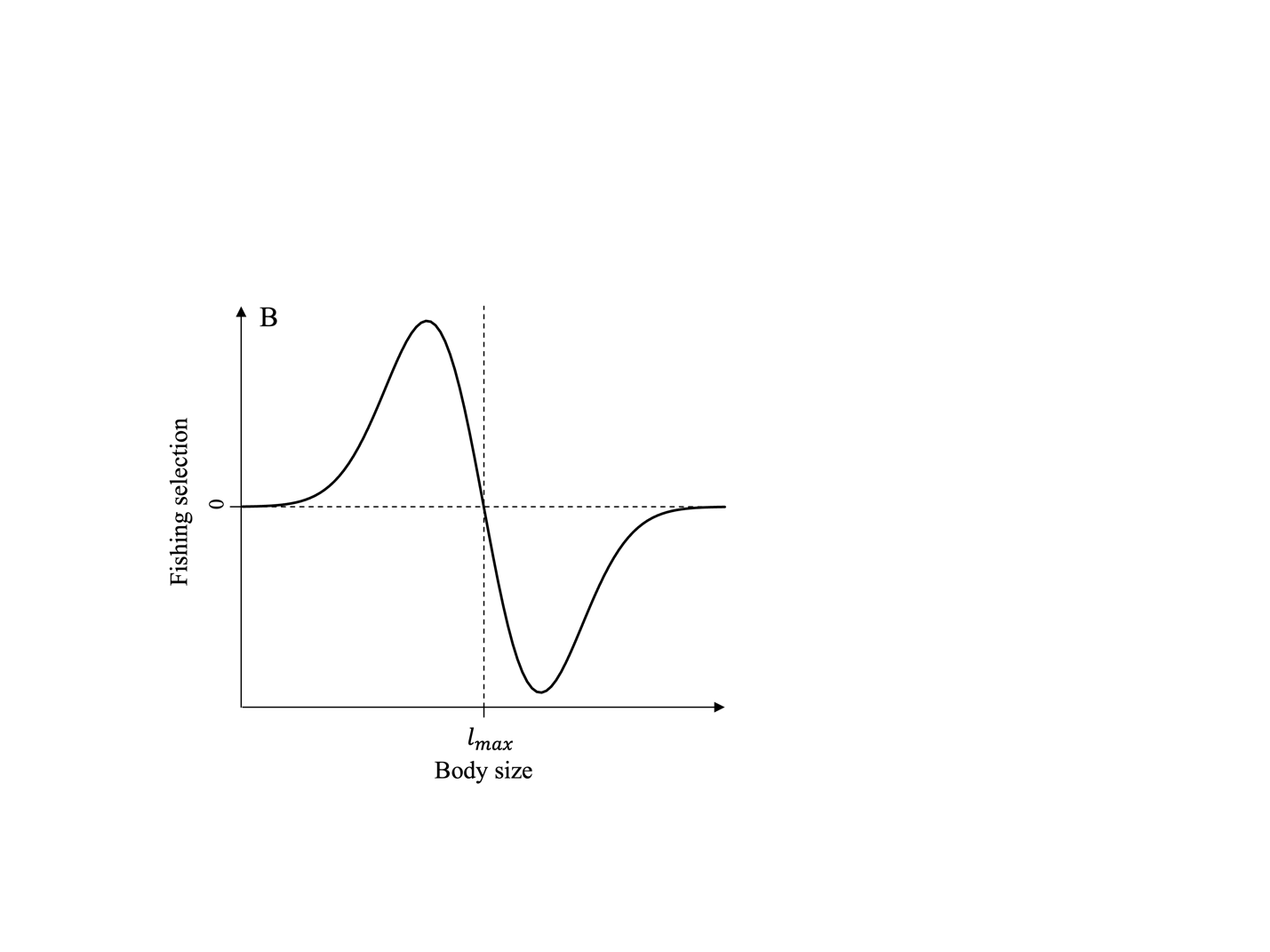


Figure S1. Fishing mortality (panel A) and fishing selection strength (panel B) under varied body size for gillnets.

# Figure S2: The dependency of $\boldsymbol{r(u)}$, $\boldsymbol{a(u,v)}$ and $\boldsymbol{g(v)}$ on traits.


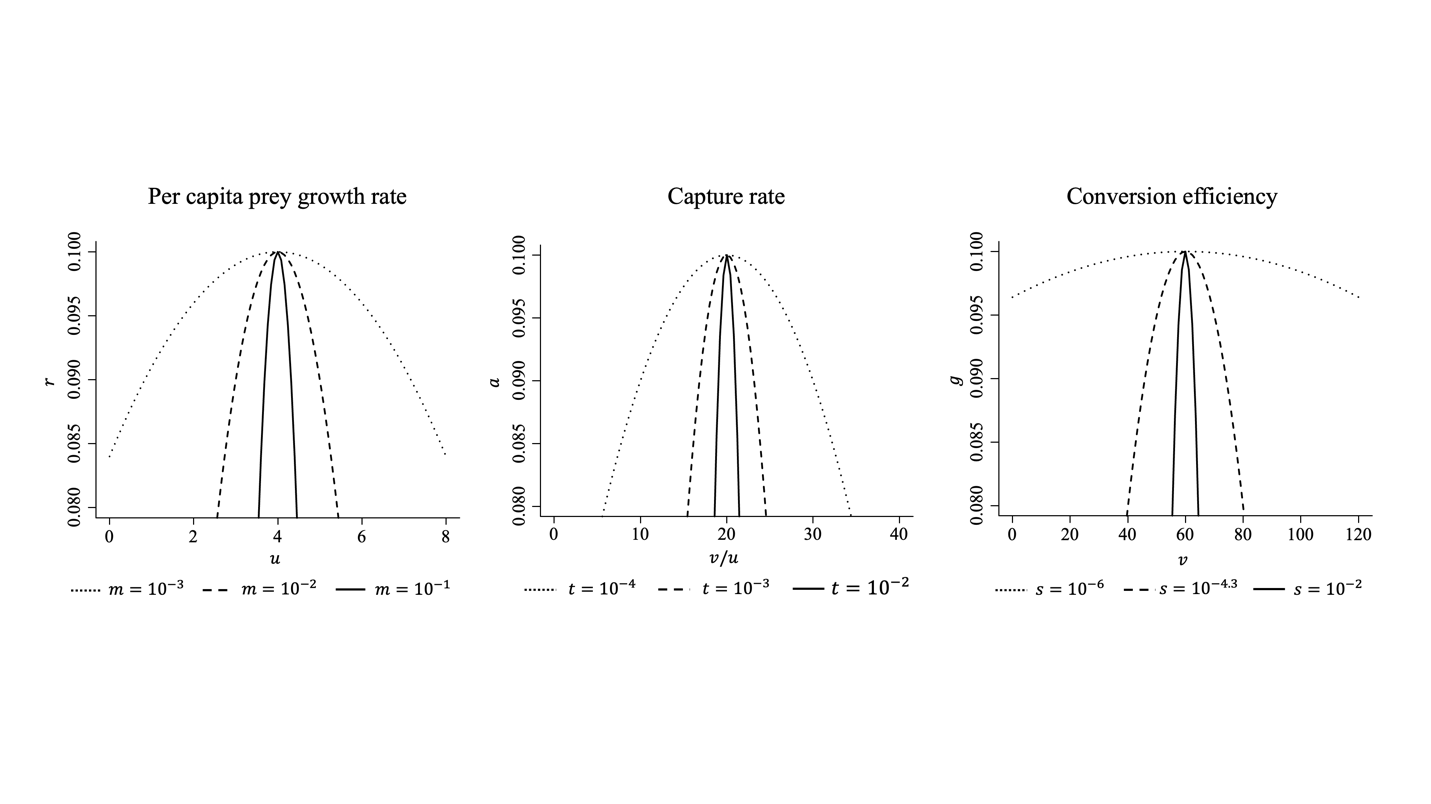


Figure S2. The curves of $r(u)$, $a(u,v)$ and $g(v)$ when $m$, $s$ and $t$ take on various values.

# Figure S3: The impact of initial value setting to the final equilibrium point and effect of prey adaptation to predator persistence in LB case.

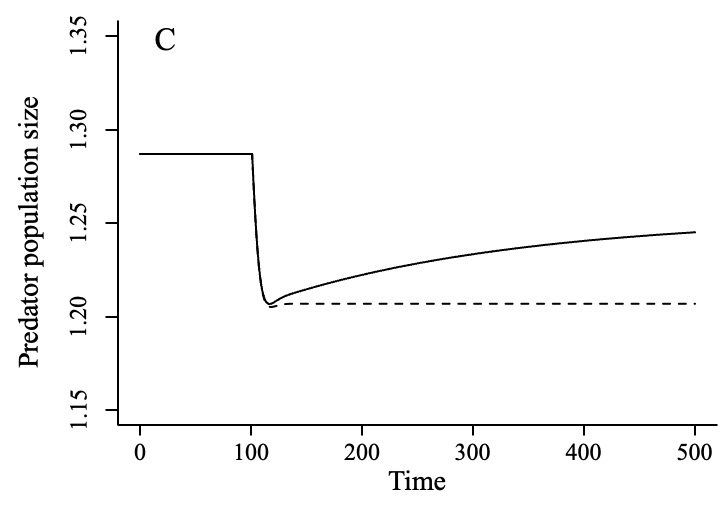


Figure S3. Two possible equilibria and rescue effect of prey adaptation in LB case. In panel A, two possible equilibrium points are shown, which are $\left\{ x_{e}^{*}=93.38,y_{e}^{*}=0.26, u_{e}^{*}=2.73, v_{e}^{*}=60.00 \right\}$ and $\left\{ x_{e}^{*}=76.52,y_{e}^{*}=1.29, u_{e}^{*}=5.28, v_{e}^{*}=60.00 \right\}$. The setting of initial value results in different equilibrium points. Panel B, the dynamics of predator population size under the scenario of non-evolvable prey and evolvable prey when the system is at equilibrium point $\left\{ x_{e}^{*}=93.38,y_{e}^{*}=0.26, u_{e}^{*}=2.73, v_{e}^{*}=60.00 \right\}$. Panel C, the dynamics of predator population size when the system is at equilibrium point $\left\{ x_{e}^{*}=76.52,y_{e}^{*}=1.29, u_{e}^{*}=5.28, v_{e}^{*}=60.00 \right\}$. The predator is harvested at time 100. Parameters setting: $c=0.005$, $d=0.6$, $g_{0}=0.1$, $a_{0}=0.1$, $r_{0}=0.5$, $m=0.01$, $t=0.001$, $u_{0}=4$, $v_{0}=60$, $R_{0}=16$, $V_{x}=0.1 or 0$ (for evolutionary and non-evolutionary scenarios, respectively), $V_{y}=0$, $H=0.01$, $l_{max}=v_{e}*1.02$, $p=10$. Initial value: {$x=60,y=12, u=3.5, v=60$} for panel B, and {$x=60,y=12, u=4.5, v=60$} for panel C.

# Appendix S1: Model framework and equilibrium points

The basic eco-evolutionary predator-prey system, where fisheries component is excluded, is described as the following:

$\frac{dx}{dt}=\left( r-cx-ay \right)x$ (A1)

$\frac{dy}{dt}=\left( gax-d \right)y$ (A2)

$\frac{du}{dt}=V_{x}(\frac{dr}{du}-\frac{\partial a}{\partial u}\cdot y)$ (A3)

$\frac{dv}{dt}=V_{y}(\frac{dg}{dv}\cdot ax+\frac{\partial a}{\partial v}\cdot gx)$ (A4)

Equations (A1) and (A2) depict ecological dynamics and Equations (A3) and (A4) denote evolutionary dynamics.

The equilibrium point will be

$x^{*}=\frac{d}{g(v^{*})a(u^{*}, v^{*})}$ (e1)

$y^{*}=\frac{r\left( u^{*} \right)}{a\left( u^{*}, v^{*} \right)}\cdot[1-\frac{cd}{g\left( v^{*} \right)a\left( u^{*}, v^{*} \right)r\left( u^{*} \right)}]$ (e2)

$\frac{dr}{du}|_{u=u^{*}}=-\frac{v^{*}}{\left( u^{*} \right)^{2}}\cdot y^{*}\cdot\frac{\partial a}{\partial(\frac{v}{u})}|_{u=u^{*} , v=v^{*}}$ (e3)

$\frac{dg}{dv}|_{v=v^{*}}=-\frac{g(v^{*})}{a\left( u^{*}, v^{*} \right)}\cdot\frac{1}{u^{*}}\cdot\frac{\partial a}{\partial(\frac{v}{u})}|_{u=u^{*} , v=v^{*}}$ (e4)

Equation (e3) and (e4) implies three possible final equilibrium states (Figure S4). In State I: $v^{*}>v_{0}$, $u^{*}>u_{0}$ and $\frac{v^{*}}{u^{*}}<R_{0}$, which make $\frac{dr}{du}|_{u=u^{*}}$ and $\frac{dg}{dv}|_{v=v^{*}}$ negative, and $\frac{da}{d(\frac{v}{u})}|_{u=u^{*}, v=v^{*}}$ positive. In State II: $v^{*}<v_{0}$, $u^{*}<u_{0}$ and $\frac{v^{*}}{u^{*}}>R_{0}$, leading to positive $\frac{dr}{du}|_{u=u^{*}}$ and $\frac{dg}{dv}|_{v=v^{*}}$, negative $\frac{da}{d(\frac{v}{u})}|_{u=u^{*}, v=v^{*}}$. In State III: $v^{*}=v_{0}$, $u^{*}=u_{0}$ and $\frac{v^{*}}{u^{*}}=R_{0}$, leading to $\frac{dr}{du}|_{u=u^{*}}=0$, $\frac{dg}{dv}|_{v=v^{*}}=0$ and $\frac{da}{d(\frac{v}{u})}|_{u=u^{*}, v=v^{*}}=0$. The State III is possible only when $R_{0}=\frac{v_{0}}{u_{0}}$.

In this study, the setting of initial value may affect coevolutionary process and result in different equilibrium points. Because our analysis is based on equilibrium states, and any equilibrium point must belong to one of these equilibrium states, the dependence of finial equilibrium point on initial value does not alter our conclusions in this study (Figure S3). Considering the understandability, we take the point ($u=u_{0}$, $v=v_{0}$) as the initial point without loss of generality. At this initial point, when $\frac{v_{0}}{u_{0}}<R_{0}$, $\frac{du}{dt}$ will be positive due to negative $\frac{da}{du}|_{u=u_{0}, v=v_{0}}$ according to equation (A3), and $\frac{dv}{dt}$ will be positive arising from the positive $\frac{da}{dv}|_{u=u_{0}, v=v_{0}}$ according to equation (A4), resulting in the State I. Correspondently, when $\frac{v_{0}}{u_{0}}>R_{0}$, $\frac{du}{dt}$ and $\frac{dv}{dt}$ will be negative, leading to the State II. When $\frac{v_{0}}{u_{0}}=R_{0}$, $\frac{du}{dt}$ and $\frac{dv}{dt}$ will be 0, leading to the State III.


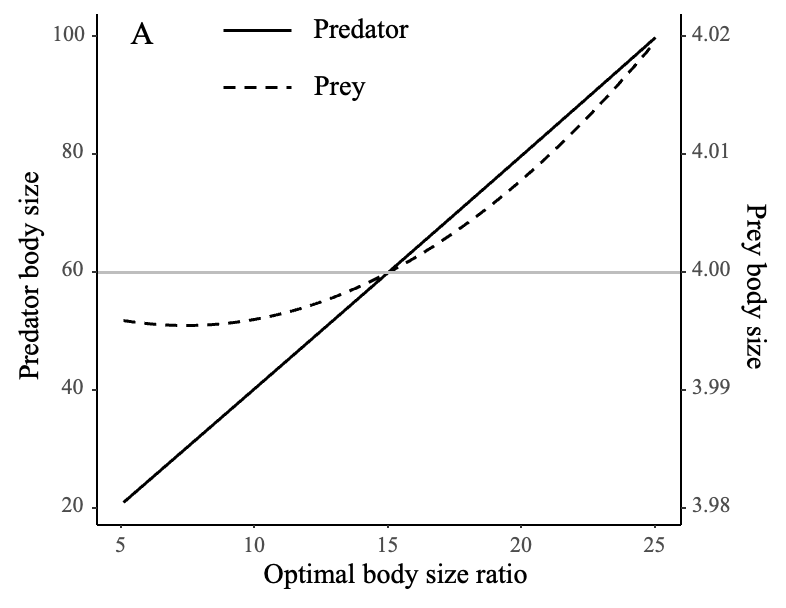

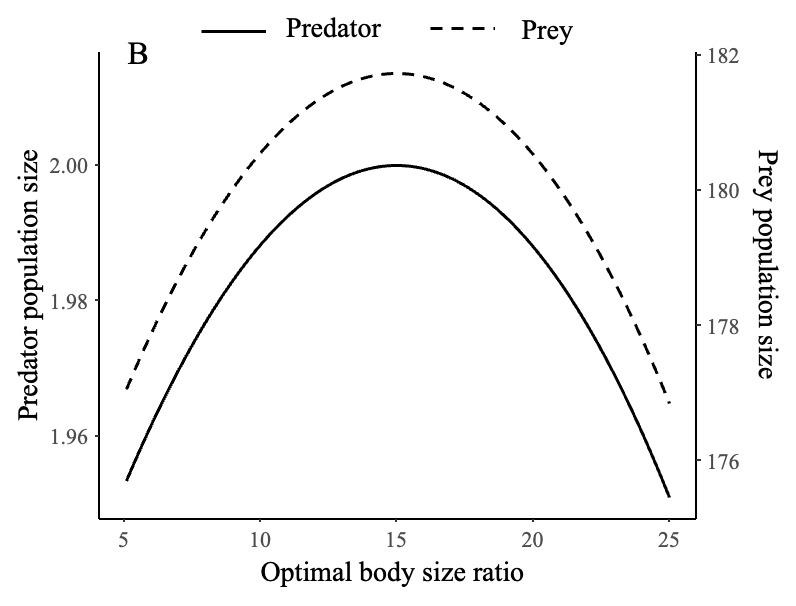


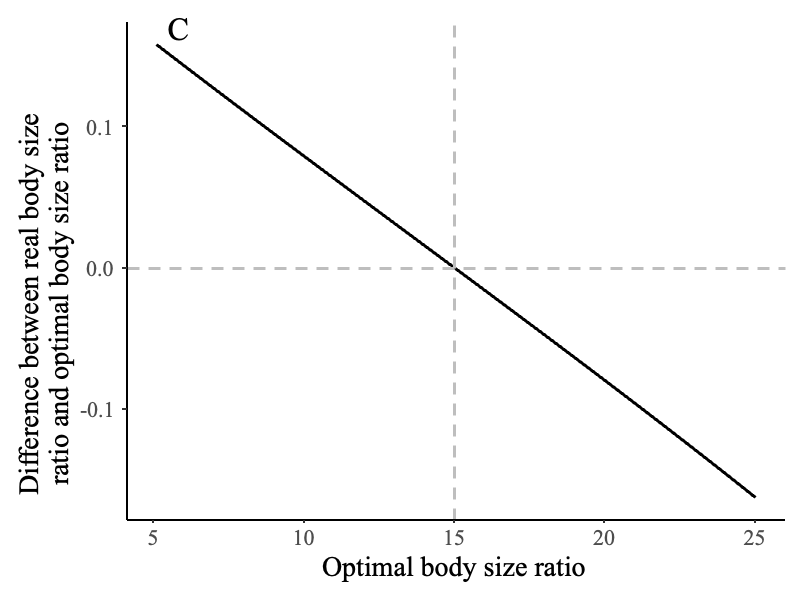


Figure S4. The equilibrium points under varied optimal body size ratio ($R_{0}$). Panel A indicates the trait values of prey and predator at equilibrium, and panel B refers to the density of prey and predator at equilibrium. Panel C denotes the difference between real body size ratio and optimal body size ratio under varied $R_{0}$. In panels A and B, black solid lines and black dash lines represent predator and prey, respectively. Gray line denotes the optimal body size for predator and prey. Parameters setting: $c=0.005$, $d=0.6$, $g_{0}=0.1$, $a_{0}=0.1$, $r_{0}=0.5$, $s={10}^{-6}, m=0.1$, $t=0.001$, $u_{0}=4$, $v_{0}=60$, $R_{0}=5\sim25$.

# Appendix S2: Stability analysis

We study the local stability of the system described by Equations (A1), (A2), (A3) and (A4) by linearizing the dynamics near the nontrivial equilibrium. We judge the local stability by whether the characteristic equation of their Jacobian matrix satisfies the Routh-Hurwitz criteria. Because of the symmetry between State I and State II, we mainly analyze the stability of the system at equilibrium State I and III.

## Stability evaluation method

The Jacobian matrix $\boldsymbol{J}$ can be obtained by evaluating the derivative of Equations (A1), (A2), (A3) and (A4) with respect to $x,y, u$ and $v$ at the equilibrium point $P^{*}$: {$x=x^{*},y=y^{*}, u=u^{*}, v=v^{*}$}, respectively.

$$\boldsymbol{J}=\left[ \begin{matrix} \begin{matrix} J_{11} & J_{12} \\ J_{21} & J_{22} \end{matrix} & \begin{matrix} J_{13} & J_{14} \\ J_{23} & J_{24} \end{matrix} \\ \begin{matrix} J_{31} & J_{32} \\ J_{41} & J_{42} \end{matrix} & \begin{matrix} J_{33} & J_{34} \\ J_{43} & J_{44} \end{matrix} \end{matrix} \right]$$

where $J_{11}=\frac{d(\frac{dx}{dt})}{dx}|_{P^{*}}$, $J_{12}=\frac{d(\frac{dx}{dt})}{dy}|_{P^{*}}$, $J_{13}=\frac{d(\frac{dx}{dt})}{du}|_{P^{*}}$, $J_{14}=\frac{d(\frac{dx}{dt})}{dv}|_{P^{*}}$, other elements in Jacobian matrix $\boldsymbol{J}$ follow the same rule as $J_{11}$, $J_{12}$, $J_{13}$ and $J_{14}$, and the formula for each element is shown in table A1.

Table A1: Jacobian matrix entries at equilibrium

| $J_{11}=r-2cx-ay$ | $J_{12}=-ax$ | $J_{13}=\left( \frac{dr}{du}-\frac{\partial a}{\partial u}\cdot y \right)x$ | $J_{14}=-\frac{\partial a}{\partial v}yx$ |
| --- | --- | --- | --- |
| $J_{21}=gay$ | $J_{22}=gax-d$ | $J_{23}=\frac{\partial a}{\partial u}gxy$ | $J_{24}=\left( \frac{dg}{dv}\cdot a+\frac{\partial a}{\partial v}\cdot g \right)xy$ |
| $J_{31}=0$ | $J_{32}=-V_{x}\frac{\partial a}{\partial u}$ | $J_{33}=V_{x}(\frac{d^{2}r}{du^{2}}-\frac{\partial^{2}a}{\partial u^{2}}y)$ | $J_{34}=V_{x}(-\frac{\partial^{2}a}{\partial u\partial v}g)y$ |
| $J_{41}=V_{y}\left( \frac{dg}{dv}a+\frac{\partial a}{\partial v}g \right)$ | $J_{42}=0$ | $J_{43}=V_{y}(\frac{dg}{dv}\frac{\partial a}{\partial u}+\frac{\partial^{2}a}{\partial u\partial v}g)x$ | $J_{44}=V_{y}(\frac{d^{2}g}{dv^{2}}a+2\frac{dg}{dv}\frac{\partial a}{\partial v}+\frac{\partial^{2}a}{\partial v^{2}}g)x$ |

Throughout, $\left| M_{(ij)(ij)} \right|$ and $\left| M_{(ijk)(ijk)} \right|$ denote determinants of the following submatrices of $\boldsymbol{J}$ evaluated at $V_{x}=V_{y}=1$,

$\left| M_{(ij)(ij)} \right|=\left| \begin{matrix} J_{ii} & J_{ij} \\ J_{ji} & J_{jj} \end{matrix} \right|$, $\left| M_{(ijk)(ijk)} \right|=\left| \begin{matrix} J_{ii} & J_{ij} & J_{ik} \\ J_{ji} & J_{jj} & J_{jk} \\ J_{ki} & J_{kj} & J_{kk} \end{matrix} \right|$.

Here, the indices in the first set of parentheses denote the rows and the indices in the second set of parentheses denote the columns used to construct the submatrix. Submatrices of $\boldsymbol{J}$ evaluated at $V_{x}=V_{y}=1$ are used to make it more clear how the terms in the Routh-Hurwitz sequence depend on $V_{x}$and $V_{y}$.

The characteristic polynomial of $\boldsymbol{J}$ is defined as

$\rho\left( \lambda\right)=\left| \boldsymbol{J}-\lambda\boldsymbol{I} \right|=\lambda^{4}+w_{1}\lambda^{3}+w_{2}\lambda^{2}+w_{3}\lambda+w_{4}$

where $\boldsymbol{I}$ is the 4$\times$4 identify matrix. The coefficients in the characteristic polynomial are

$$w_{1}=-\left( J_{11}+J_{22} \right)-V_{x}J_{33}-V_{y}J_{44}$$

$$w_{2}=\left| M_{(12)(12)} \right|+V_{x}\left( \left| M_{\left( 13 \right)\left( 13 \right)} \right|+\left| M_{\left( 23 \right)\left( 23 \right)} \right| \right)+V_{y}\left( \left| M_{\left( 14 \right)\left( 14 \right)} \right|+\left| M_{\left( 24 \right)\left( 24 \right)} \right| \right)+V_{x}V_{y}\left| M_{\left( 34 \right)\left( 34 \right)} \right|$$

$$w_{3}=-V_{x}\left| M_{\left( 123 \right)\left( 123 \right)} \right|-V_{y}\left| M_{\left( 124 \right)\left( 124 \right)} \right|-V_{x}V_{y}\left( \left| M_{\left( 134 \right)\left( 134 \right)} \right|+\left| M_{\left( 234 \right)\left( 234 \right)} \right| \right)$$

$$w_{4}=\left| \boldsymbol{J} \right|$$

From the Routh-Hurwitz stability criteria, the stability conditions will be: $w_{1}>0$, $w_{1}(w_{1}w_{2}-w_{3})>0$, $w_{3}-\frac{w_{1}^{2}w_{4}}{w_{1}w_{2}-w_{3}}>0$ and $w_{4}>0$.

## Stability conditions

Considering that the system is four-dimensional and nonlinear, as well as the dependence of specific equilibrium point on parameter setting, it is difficult to get the general analytical stability condition. While, because of the uniqueness of equilibrium point in the UB case from State I, {$x=x^{*},y=y^{*}, u=u_{0}, v=R_{0}v_{0}$}, and equilibrium point in State III, {$x=x^{*},y=y^{*}, u=u_{0}, v=v_{0}$}, the analytical stability condition can be obtained.

At the equilibrium point {$x=x^{*},y=y^{*}, u=u_{0}, v=R_{0}v_{0}$} in the UB case from State I, or {$x=x^{*},y=y^{*}, u=u_{0}, v=v_{0}$} in State III, Jacobian matrix $\boldsymbol{J}$ can be obtained, which is

$$\boldsymbol{J}=\left\{ \begin{matrix} \begin{matrix} J_{11} & J_{12} \\ J_{21} & 0 \end{matrix} & \begin{matrix} 0 & 0 \\ 0 & 0 \end{matrix} \\ \begin{matrix} 0 & 0 \\ 0 & 0 \end{matrix} & \begin{matrix} J_{33} & J_{34} \\ J_{43} & J_{44} \end{matrix} \end{matrix} \right\}$$

where $J_{11}=-cx^{*}$, $J_{12}=-a_{0}y^{*}$, $J_{21}=g_{0}a_{0}y^{*}$, $J_{33}=(-2m+\frac{2tR_{0}^{2}}{u_{0}^{2}}y^{*})V_{x}$, $J_{34}=(-\frac{2tR_{0}}{u_{0}^{2}}y^{*})V_{x}$, $J_{43}=\frac{2tR_{0}}{u_{0}^{2}}g_{0}x^{*}V_{y}$, $J_{44}=-\frac{2t}{u_{0}^{2}}g_{0}x^{*}V_{y}$. Due to elements in submatrices of $\left( \begin{matrix} J_{13} & J_{14} \\ J_{23} & J_{24} \end{matrix} \right)$ and $\left( \begin{matrix} J_{31} & J_{32} \\ J_{41} & J_{42} \end{matrix} \right)$ are all 0, there is no eco-evolutionary feedback loop, and the system stability depends on the stability of ecological subsystem $\left( \begin{matrix} J_{11} & J_{12} \\ J_{21} & J_{22} \end{matrix} \right)$ and the stability of evolutionary subsystem $\left( \begin{matrix} J_{33} & J_{34} \\ J_{43} & J_{44} \end{matrix} \right)$ (Cortez 2018).

In the ecological subsystem, because of $w_{1}=-\left( J_{11}+J_{22} \right)=cx^{*}>0$, $w_{2}=J_{11}J_{22}-J_{12}J_{21}=g_{0}{(a_{0}y^{*})}^{2}>0$, the ecological subsystem is always stable.

In the evolutionary subsystem,

$$-(J_{33}+J_{44})=\frac{2t}{u_{0}^{2}}\left( g_{0}x^{*}V_{y}-R_{0}^{2}y^{*}V_{x} \right)+2mV_{x}$$

$$J_{33}J_{34}-J_{34}J_{43}=\frac{4tm}{u_{0}^{2}}g_{0}x^{*}V_{x}V_{y}$$

From $-\left( J_{33}+J_{44} \right)>0$, we can get

$$\frac{V_{y}}{V_{x}}>\frac{R_{0}^{2}\cdot y^{*}-m*\frac{u_{0}^{2}}{t}}{g_{0}\cdot x^{*}}$$

And $J_{33}J_{34}-J_{34}J_{43}>0$ always holds.

Therefore, the stability condition for the whole system will be

$$\frac{V_{y}}{V_{x}}>\frac{R_{0}^{2}\cdot y^{*}-m*\frac{u_{0}^{2}}{t}}{g_{0}\cdot x^{*}}$$

According to the parameter settings in these two special situations, stability condition in the UB case will be (Figure S5)

$$\frac{V_{y}}{V_{x}}>\frac{400-8*\frac{m}{t}}{3}$$

And stability condition in State III will be (Figure S6)

$$\frac{V_{y}}{V_{x}}>\frac{225-8*\frac{m}{t}}{3}$$


Figure S5. Parameters region in which the system is stable at equilibrium point in the UB case. The two axes are $V_{x}$ and $V_{x}$. Parameter setting: $m=0.01$. The white and shaded regions are the regions in which the equilibrium is stable and unstable, respectively. As $t$ increases, the stable region (left part of the line) gradually shrinks.

Figure S6. Parameters region in which the system is stable at equilibrium point in equilibrium State (III). The two axes are $V_{x}$ and $V_{x}$. Parameter setting: $m=0.01$. The white and shaded regions are the regions in which the equilibrium is stable and unstable, respectively. As $t$ increases, the stable region (left part of the line) gradually shrinks.

Reference

Cortez, M.H. (2018). Genetic variation determines which feedbacks drive and alter predator-prey eco-evolutionary cycles. *Ecological Monographs*, 88(3), 353-371.
